# 16S rRNA amplicon sequencing of bacterial communities from stormwater ponds in South Florida

**DOI:** 10.1128/mra.00690-25

**Published:** 2025-08-25

**Authors:** Jéssica A. S. Moretto, Forrest W. Lefler, Meng Bing Lin, David E. Berthold, Maximiliano Barbosa, Ashley R. Smyth, H. Dail Laughinghouse

**Affiliations:** 1 Agronomy Department, Fort Lauderdale Research and Education Center, Institute of Food and Agricultural Sciences, University of Florida 53701 , Davie, Florida, USA; 2 Soil, Water & Ecosystem Sciences Department, Tropical Research and Education Center, Institute of Food and Agricultural Sciences, University of Florida 53701, Homestead, Florida, USA; DOE Joint Genome Institute, Berkeley, California, USA

**Keywords:** cyanobacteria, eubacteria, aquatic microbiology, amplicon sequencing

## Abstract

Stormwater ponds (SWPs) host distinct microbial communities. Here, we use 16S rRNA metabarcoding to characterize bacterial communities in four SWPs in South Florida, USA. A total of 37,002 amplicon sequence variants were identified, Cyanophyceae was the dominant class in pond A, while Gammaproteobacteria dominated the other ponds.

## ANNOUNCEMENT

**Fig 1:**
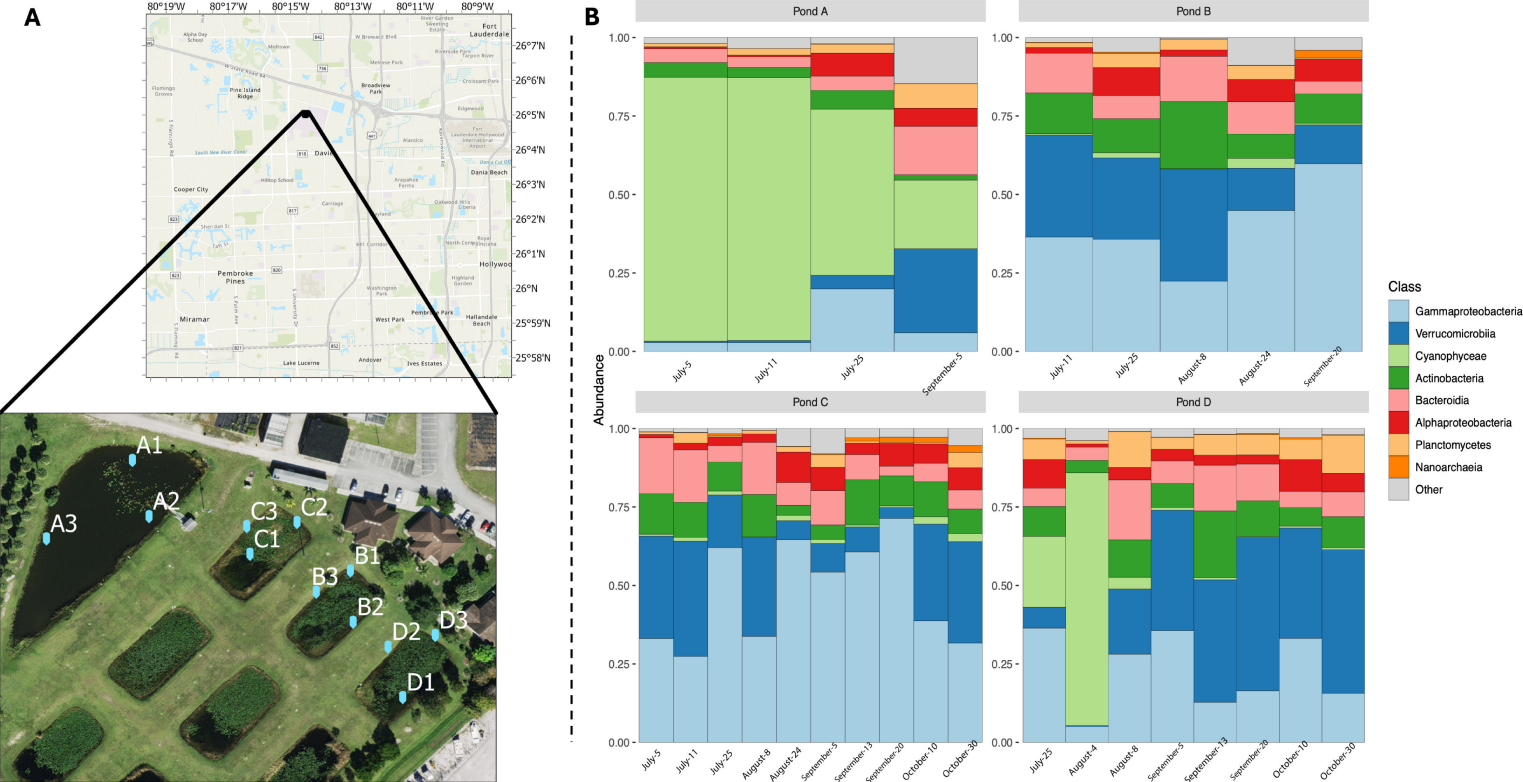
(**A**). Sample site locations in Davie, Florida, USA. (**B**) Relative abundance of bacterial communities at the class level across pond samples. Average of the three replicate samples collected from each pond.

Stormwater ponds (SWPs) are engineered ecosystems designed for flood control by storing stormwater runoff (1). In addition to their hydrological function, these systems are “hot spots” for biogeochemical processes (e.g., nitrogen cycling) and host distinct and dynamic microbial communities that are important for biogeochemical processes (2, 3). Despite their ecological relevance, information on bacterial communities in SWPs remains limited. Here, we report 16S rRNA gene sequencing data of SWP microbial communities sampled from four SWPs in Davie, Florida, USA (Fig. 1A; Table 1).

**TABLE 1:** Information of samples and amplicon reads

Sample name	Pond	Date	Rep	# GenBank accession	GPS LAT	GPS LONG	# Reads	# ASV
NB1.raw	Pond A	5-July-23	1	SRR33984429	26°05′02.3″N	80°14′33.1″W	105,718	791
NB2.raw	Pond A	5-July 23	2	SRR33984428	26°05′01.6″N	80°14′32.9″W	106,668	672
NB3.raw	Pond A	5-July-23	3	SRR33984453	26°05′01.2″N	80°14′34.3″W	102,094	1,263
NB7.raw	Pond A	11-July-23	1	SRR33984442	26°05′02.3″N	80°14′33.1″W	86,538	973
NB8.raw	Pond A	11-July-23	2	SRR33984431	26°05′01.6″N	80°14′32.9″W	106,462	867
NB9.raw	Pond A	11-July-23	3	SRR33984416	26°05′01.2″N	80°14′34.3″W	102,015	961
NB16.raw	Pond A	25-July-23	1	SRR33984405	26°05′02.3″N	80°14′33.1″W	88,089	2,745
NB17.raw	Pond A	25-July-23	2	SRR33984394	26°05′01.6″N	80°14′32.9″W	109,732	1,433
NB18.raw	Pond A	25-July-23	3	SRR33984389	26°05′01.2″N	80°14′34.3″W	105,182	1,314
NB34.raw	Pond A	5-September-23	1	SRR33984388	26°05′02.3″N	80°14′33.1″W	104,820	2,555
NB36.raw	Pond A	5-September-23	3	SRR33984427	26°05′01.6″N	80°14′32.9″W	79,138	3,063
NB19.raw	Pond B	25-July-23	1	SRR33984426	26°05′00.8″N	80°14′30.2″W	105,316	3,787
NB20.raw	Pond B	25-July-23	2	SRR33984461	26°05′00.1″N	80°14′30.2″W	119,145	2,562
NB21.raw	Pond B	25-July-23	3	SRR33984460	26°05′00.5″N	80°14′30.7″W	105,925	3,496
NB73.raw	Pond B	8-August-23	1	SRR33984459	26°05′00.8″N	80°14′30.2″W	106,647	1,204
NB74.raw	Pond B	8-August-23	2	SRR33984458	26°05′00.1″N	80°14′30.2″W	103,806	1,259
NB75.raw	Pond B	8-August-23	3	SRR33984457	26°05′00.5″N	80°14′30.7″W	108,118	1,055
NB28.raw	Pond B	23-August-23	1	SRR33984456	26°05′00.8″N	80°14′30.2″W	103,941	3,224
NB29.raw	Pond B	23-August-23	2	SRR33984455	26°05′00.1″N	80°14′30.2″W	106,212	6,707
NB30.raw	Pond B	23-August-23	3	SRR33984454	26°05′00.5″N	80°14′30.7″W	102,149	4,450
NB49.raw	Pond B	20-September-23	1	SRR33984452	26°05′00.8″N	80°14′30.2″W	104,080	2,459
NB50.raw	Pond B	20-September-23	2	SRR33984451	26°05′00.1″N	80°14′30.2″W	107,702	2,329
NB51.raw	Pond B	20-September-23	3	SRR33984450	26°05′00.5″N	80°14′30.7″W	102,116	2,819
NB4.raw	Pond C	5-July-23	1	SRR33984449	26°05′01.0″N	80°14′31.5″W	105,088	2,206
NB5.raw	Pond C	5-July-23	2	SRR33984448	26°05′01.5″N	80°14′30.9″W	106,426	1,497
NB6.raw	Pond C	5-July-23	3	SRR33984447	26°05′01.4″N	80°14′31.6″W	108,570	1,001
NB10.raw	Pond C	11-July-23	1	SRR33984446	26°05′01.0″N	80°14′31.5″W	105,780	2,010
NB11.raw	Pond C	11-July-23	2	SRR33984445	26°05′01.5″N	80°14′30.9″W	115,224	1,528
NB12.raw	Pond C	11-July-23	3	SRR33984444	26°05′01.4″N	80°14′31.6″W	104,074	1,684
NB22.raw	Pond C	25-July-23	1	SRR33984443	26°05′01.0″N	80°14′31.5″W	103,220	2,283
NB24.raw	Pond C	25-July-23	3	SRR33984441	26°05′01.5″N	80°14′30.9″W	105,139	1,938
NB76.raw	Pond C	8-August-23	1	SRR33984440	26°05′01.4″N	80°14′31.6″W	102,070	1,332
NB77.raw	Pond C	8-August-23	2	SRR33984439	26°05′01.0″N	80°14′31.5″W	105,899	1,502
NB78.raw	Pond C	8-August-23	3	SRR33984438	26°05′01.5″N	80°14′30.9″W	109,573	1,408
NB32.raw	Pond C	24-August-23	2	SRR33984437	26°05′01.4″N	80°14′31.6″W	106,736	3,715
NB33.raw	Pond C	24-August-23	3	SRR33984436	26°05′01.0″N	80°14′31.5″W	102,104	4,312
NB37.raw	Pond C	24-August-23	1	SRR33984435	26°05′01.5″N	80°14′30.9″W	116,665	5,238
NB39.raw	Pond C	5-September-23	3	SRR33984434	26°05′01.4″N	80°14′31.6″W	105,552	3,979
NB43.raw	Pond C	13-September-23	1	SRR33984433	26°05′01.0″N	80°14′31.5″W	103,886	2,172
NB44.raw	Pond C	13-September-23	2	SRR33984432	26°05′01.5″N	80°14′30.9″W	102,507	2,395
NB45.raw	Pond C	13-September-23	3	SRR33984430	26°05′01.4″N	80°14′31.6″W	103,250	2,124
NB52.raw	Pond C	20-September-23	1	SRR33984425	26°05′01.0″N	80°14′31.5″W	116,088	2,089
NB53.raw	Pond C	20-September-23	2	SRR33984424	26°05′01.5″N	80°14′30.9″W	95,262	1,871
NB54.raw	Pond C	20-September-23	3	SRR33984423	26°05′01.4″N	80°14′31.6″W	102,144	2,490
NB58.raw	Pond C	10-October-23	1	SRR33984422	26°05′01.0″N	80°14′31.5″W	102,139	1,900
NB59.raw	Pond C	10-October-23	2	SRR33984421	26°05′01.5″N	80°14′30.9″W	104,854	2,279
NB60.raw	Pond C	10-October-23	3	SRR33984420	26°05′01.4″N	80°14′31.6″W	66,508	2,226
NB64.raw	Pond C	30-October-23	1	SRR33984419	26°05′01.0″N	80°14′31.5″W	102,319	2,672
NB65.raw	Pond C	30-October-23	2	SRR33984418	26°05′01.5″N	80°14′30.9″W	103,182	4,180
NB66.raw	Pond C	30-October-23	3	SRR33984417	26°05′01.4″N	80°14′31.6″W	102,067	4,274
NB79.raw	Pond D	8-August-23	1	SRR33984415	26°04′59.1″N	80°14′29.5″W	105,362	1,467
NB80.raw	Pond D	8-August-23	2	SRR33984414	26°05′00.0″N	80°14′29.7″W	106,220	1,519
NB81.raw	Pond D	8-August-23	3	SRR33984413	26°05′00.0″N	80°14′29.1″W	103,879	1,437
NB40.raw	Pond D	5-September-23	1	SRR33984412	26°04′59.1″N	80°14′29.5″W	104,951	4,161
NB41.raw	Pond D	5-September-23	2	SRR33984411	26°05′00.0″N	80°14′29.7″W	106,640	2,839
NB42.raw	Pond D	5-September-23	3	SRR33984410	26°05′00.0″N	80°14′29.1″W	112,501	1,460
NB46.raw	Pond D	13-September-23	1	SRR33984409	26°04′59.1″N	80°14′29.5″W	105,813	3,652
NB47.raw	Pond D	13-September-23	2	SRR33984408	26°05′00.0″N	80°14′29.7″W	105,325	1,958
NB48.raw	Pond D	13-September-23	3	SRR33984407	26°05′00.0″N	80°14′29.1″W	102,947	1,314
NB55.raw	Pond D	20-September-23	1	SRR33984406	26°04′59.1″N	80°14′29.5″W	112,071	1,703
NB56.raw	Pond D	20-September-23	2	SRR33984404	26°05′00.0″N	80°14′29.7″W	105,007	1,734
NB57.raw	Pond D	20-September-23	3	SRR33984403	26°05′00.0″N	80°14′29.1″W	102,110	1,997
NB61.raw	Pond D	10-October-23	1	SRR33984402	26°04′59.1″N	80°14′29.5″W	104,516	2,082
NB62.raw	Pond D	10-October-23	2	SRR33984401	26°05′00.0″N	80°14′29.7″W	105,842	3,379
NB63.raw	Pond D	10-October-23	3	SRR33984400	26°05′00.0″N	80°14′29.1″W	105,306	1,641
NB67.raw	Pond D	30-October-23	1	SRR33984399	26°04′59.1″N	80°14′29.5″W	103,201	1,721
NB68.raw	Pond D	30-October-23	2	SRR33984398	26°05′00.0″N	80°14′29.7″W	103,519	3,522
NB69.raw	Pond D	30-October-23	3	SRR33984397	26°05′00.0″N	80°14′29.1″W	103,276	1,382
NB25.raw	Pond D	25-July-23	1	SRR33984396	26°04′59.1″N	80°14′29.5″W	78,177	1,027
NB26.raw	Pond D	25-July-23	2	SRR33984395	26°05′00.0″N	80°14′29.7″W	105,242	2,937
NB27.raw	Pond D	25-July-23	3	SRR33984393	26°05′00.0″N	80°14′29.1″W	106,329	3,683
NB70.raw	Pond D	4-August-23	1	SRR33984392	26°04′59.1″N	80°14′29.5″W	103,623	962
NB71.raw	Pond D	4-August-23	2	SRR33984391	26°05′00.0″N	80°14′29.7″W	105,229	840
NB72.raw	Pond D	4-August-23	3	SRR33984390	26°05′00.0″N	80°14′29.1″W	106,454	913

Independent sub-surface water samples were collected using acid-washed and sterile 1 L Nalgene bottles, to characterize the water column microbial community structure. A total of 74 samples were collected between July and October 2023 (Table 1). Water samples were filtered onto a 0.22 µm MCE filter (Millipore Sigma, Darmstadt, Germany) until clogging (~1 L). DNA was extracted from the filters using a Qiagen Blood Tissue kit with modified protocols by Djurhuus et al. (4). The extracted DNA was used for bacterial community analysis via amplification of the V3–V4 region of 16S rRNA using the primers 515F-Y (5′-GTGYCAGCMGCCGCGGTAA) and 926R (5′-CCGYCAATTYMTTTRAGTTT) according to Parada et al. (5). Library preparation was performed using Illumina Nextera XT Index Kit v2 following the manufacturer’s protocols. The libraries were normalized and pooled according to Illumina’s recommended protocol. Sequencing was performed on an Illumina NovaSeq 6000 (paired-end 2 × 250 bp) by Novogene (Beijing, People's Republic of China). Raw Illumina reads were quality controlled with FastP V1.0 (6) and clustered into amplicon sequence variants (ASVs) using DADA2 V1.18 (7) in R v4.0.0 (R Core Team). Taxonomic assignment of ASVs was based on a naive Bayesian classification method (8) and the 16S rRNA database CyanoSeq V1.3 (9) with SILVA 138.1 (10) used as reference. Archaeal, chloroplast, eukaryotic, and mitochondrial ASVs were removed prior to downstream analyses. All tools were run with default parameters unless otherwise specified.

The number of reads per sample varied from 66,508 to 119,145, indicating variability in sequencing depth across samples. Detailed information is shown in Table 1. Cyanophyceae was the most dominant class in pond A, while Gammaproteobacteria and Verrucomicrobia dominated in other ponds (Fig. 1B). Despite the close proximity (<50 m) and identical climatic conditions, there was great variability between these ponds, highlighting the heterogeneity in microbial communities.

## Data Availability

16S rRNA amplicon DNA sequences from this study have been uploaded to the NCBI and Sequence Read Archive (SRA) under the accession number PRJNA1276527. Individual library identifiers are listed in Table 1.
